# Effectiveness of mindful self-compassion therapy on psychopathology symptoms, psychological distress and life expectancy in infertile women treated with in vitro fertilization: a two-arm double-blind parallel randomized controlled trial

**DOI:** 10.1186/s12888-023-05411-6

**Published:** 2024-03-01

**Authors:** Kimia Sahraian, Hamed Abdollahpour Ranjbar, Bahia Namavar Jahromi, Ho Nam Cheung, Joseph Ciarrochi, Mojtaba Habibi Asgarabad

**Affiliations:** 1 Department of Psychology, Higher Education Center of Eghlid, Eghlid, Iran; 2 Infertility Research Centre, Shiraz University of Medical Sciences, Shiraz, Iran; 3 Department of Psychology, Koç University College of Social Sciences and Humanities, Istanbul, Turkey; 4 Department of Obstetrics and Gynecology, School of Medical Science, Shiraz University of Medical Science, Shiraz, Iran; 5 Department of Social Work and Social Administration, The University of Hong Kong, Hong Kong SAR, China; 6 Institute for Positive Psychology and Education, Australian Catholic University, North Sydney, NSW Australia; 7 Department of Psychology, Norwegian University of Science and Technology, 7491 Dragvoll, Trondheim, Norway; 8 Health Promotion Research Center, Iran University of Medical Sciences, Tehran, Iran; 9 Department of Health Psychology, School of Behavioral Sciences and Mental Health (Tehran Institute of Psychiatry), Iran University of Medical Sciences, Tehran, Iran

**Keywords:** Mindfulness-based intervention, Self-compassion, Infertility, Psychological distress, In vitro fertilization

## Abstract

**Objectives:**

Infertility is a prominent problem affecting millions of couples worldwide. Recently, there has been a hightened emphasis on elucidating the subtle linkages between infertility treatment leveraging assisted reproductive technology and the complex realm of psychological challenges, as well as efforts in implementation of psychological interventions.The Mindful Self-Compassion (MSC) program seeks to improve self-compassion, compassion for others, mindfulness, and life satisfaction while reducing depression, anxiety, and stress. In the current study, an MSC intervention was performed on infertile women (IW) undergoing in vitro fertilization (IVF) to assess the effectiveness of this intervention in reducing psychological distress and psychopathological symptoms and enhancing life expectancy.

**Methods:**

Fifty-seven IW undergoing IVF were randomly allocated to two groups: MSC (*n* = 29) or treatment as usual (TAU; *n* = 28). Participants in MSC met once a week for two hours for eight weeks and attended a half-day meditation retreat. The Synder's Hope questionnaire and the Revised 90-Symptom Checklist (SCL-90-R) were used as the primary outcome measures. Data were obtained before the intervention, immediately after the intervention, and two months post-intervention. Repeated measures of ANCOVA and paired t-tests in all assessment points were used to compare the MSC and the TAU groups in outcomes.

**Results:**

In the MSC group, hopelessness, anger-hostility, anxiety, interpersonal sensitivity difficulties, and depression were significantly reduced compared with the TAU group, and those improvements persisted at the two-month follow-up. Reliable change index revealed that the MSC group's gains were both clinically significant and durable.

**Conclusions:**

MSC can facilitate higher life satisfaction and mental well-being for IW undergoing IVF by reducing psychological distress, psychopathological symptoms, and hopelessness. These encouraging findings call for more research into the effectiveness of mindfulness-based therapies in addressing psychological problems among IW undergoing IVF.

## Background

As per the World Health Organization (WHO), infertility has been associated with a possible contribution to disability through functional limitations, which can impact a person's overall well-being [1, 2]. As a serious life challenge, infertility can incur a disproportionate emotional burden similar to a traumatic experience and can be incredibly distressing for women [3]. Infertile patients receiving In Vitro fertilization (IVF) therapy face significant psychological distress due to their infertility, demanding medical interventions, expensive expenditures, and unexpected results [4]. Infertility among women of reproductive age is expected to afflict one in seven couples in Western nations and one in four couples in underdeveloped countries [5]. According to the WHO's recent report, 48 million couples and 186 million people globally struggle with infertility [6], with a prevalence rate of 5.0% and 2.0% for primary and secondary infertility among the Iranian population, respectively [7].

Infertility places significant emotional and social pressures on couples as well as on their social interactions. This mental distress may be exacerbated by the IVF process. As a result, the emotional state of infertile patients throughout the IVF cycle has been labeled an "emotional rollercoaster" [8], resonating with its volatility, instability, and susceptibility. On par with these adverse effects, it is found that psychological distress among these patients is significantly associated with marital instability [9]. Although infertility is not a substantial psychological disorder, it can nevertheless cause serious problems with well-being and mental health, such as depression, emotional distress, and financial challenges [10, 11]. Also, adverse emotions comprising anger, guilt, anxiety, sadness, and depression may arise among infertile couples, as well as feelings of hopelessness along with poor self-esteem and self-confidence [12–14]. According to the study by Aanchal and Deepti [15], isolation and reduced quality of life are other possible negative consequences that are frequently experienced by infertile women, in addition to anger and interpersonal antagonism [15]. Hajizade-Valokolaee et al. [16] found that there is heightened domestic violence against women undergoing IVF and higher divorce rates, which impacts treatment success rates and other indicators of well-being [16]. Several studies have found that the infertile population has a greater incidence of mental problems, such as increased depressive and anxious symptoms [17–19], higher levels of hopelessness [20–22], alexithymia [23–25], interpersonal problems [26, 27], and lower levels of quality of life [28–30]. Unfortunately, few established therapies are available for women facing infertility-related distress [31].

Infertility facilities should deliver psychosocial and emotional support to patients according to the European Society of Human Reproduction and Embryology (ESHRE) Guidelines [32] and Guidelines for Counseling in Infertility [33]. There has been a recent surge in research on psychological interventions for IVF patients. In a systematic review, Chu et al. (2017) found that nonpharmacological interventions significantly reduced negative emotions and anxiety among IVF patients [34]. Consistently, Ying et al. [35] concluded that there is utility in implementing psychosocial treatments among women and men undergoing IVF, leading to better marital function [35]. These interventions are likely to be essential, especially during the mentally taxing period of waiting for the pregnancy test results and after failed cycles [11]. Of particular import, in their assessment of psychotherapies for IVF patients, De Liz and Strauss [36] reported that group and individual/couple psychotherapy reduced anxiety and depression symptoms which were preserved in six months of follow-up [36]. The findings indicate the favorable effects of psychotherapy for infertile individuals. However, others indicate little benefit for psychosocial interventions [37]. As a result, more study into the effectiveness of psychological treatments and their incorporation into the treatment protocol for infertility conditions is necessary [38]. The present study focused on one particularly understudied question: Can an intervention that promotes self-compassion and mindfulness improve the well-being of women undergoing IVF?

## Self-compassion and the mindful self-compassion program

Kabat-Zinn defined mindfulness as “ the awareness that arises from paying attention, on purpose, in the present moment and non-judgmentally (p. 24)” [39]. And it is increasingly being employed and has been shown to be effective in a variety of health sectors [40–44]. In recent years, mindfulness research has gained attraction in Iran [45], and specific attention has been concentrated on intervention studies for individuals suffering from related conditions (e.g., infertility, menopausal difficulties, etc.), with encouraging results [46–48]. Some promising research suggests that mindfulness therapies might lessen depression and anxiety symptoms (For a review, see [49, 50]) and also other forms of psychopathology [51]. Nevertheless, few studies have delved into mindfulness-based programs (MBPs) to help infertile patients with negative emotions. A seminal clinical trial involving eight cases and no control subjects found that eight weeks of mindfulness-based cognitive therapy (MBCT) enhanced the well-being and psychological distress of infertile women (IW) [52]. Galhardo et al. [53] allocated IW to the MBP in a controlled clinical study. Program participants reported a significant decrease in depressed mood, inner and outward shame, feelings of entrapment, and failure [53]. Also, improvements in mindfulness and self-efficacy in dealing with infertility were shown to be statistically significant. Another non-randomized controlled study found that IW who participated in the intervention (i.e., mindfulness-based intervention) improved significantly in self-compassion, coping strategies based on meaning-making, mindfulness, and all fertility quality of life categories. They, moreover, revealed a considerable amelioration in emotional dysregulation strategies and active/passive avoidance coping behaviors [54].

In the context of coping with infertility and its repercussions, self-compassion as an emotion regulation strategy has been demonstrated to be highly relevant, and new research indicates that this strategy may be a milestone for future research and intervention. Cunha et al. [55] demonstrated that infertile couples, and notably women, utilize fewer emotional coping skills like self-compassion and implement more experiential avoidance and self-critical strategies [55]. In another study, Raque-Bogdan & Hoffman [56] identified that among women with primary or secondary infertility, self-compassion mediated both the relationship between the need for parenthood and subjective well-being as well as the relation between social concern and subjective well-being [56]. This finding may be especially significant given the social marginalization and stigmatized social identity of childlessness of those who are dealing with infertility in whom self-compassion can be utilized as a strategy of emotional regulation and resilience when facing self- or other-imposed blame for infertility. Also, a related study revealed that self-compassion plays a unique function in the interaction between shame and stress imposed on by infertility in both men and women [53].

The Mindful Self-Compassion Program (MSC; [57]) is a program that combines mindfulness and self-compassion and can be employed by the general public as well as clinical patients. The term "mindful" is included in the MSC program's name since it promotes principal mindfulness capabilities, which, as previously indicated, are vital to qualify for self-compassion. Participants in MSC meet once a week for two hour for eight weeks, as well as attend a half-day meditation retreat, as per the Mindfulness-Based Stress Reduction (MBSR) intervention. It should be considered that the MSC program puts emphasis on assisting participants in developing self-compassion, with mindfulness as an ancillary focus (The eight-week program devotes just one session to the cultivation of mindfulness techniques). This shows that the MSC program is a good complement to MBSR or MBCT, which have more time to spend establishing a thorough grasp of mindfulness. MSC teaches self-compassion in both formal (meditation) and casual (everyday living) settings. Each MSC session includes experiential exercises, group discussions, and homework assignments to help individuals learn how to be compassionate to themselves. The objective is to equip participants with a range of methods to help them enhance self-compassion, which they may incorporate into their daily lives as they see fit. The program also promotes broad loving-kindness skills, which are acts of friendly benevolence performed on oneself in ordinary settings [57].

Considering the emerging and promising results but a dearth of MBPs in the mental well-being of IW undergoing IVF, this study tries to underlie the knowledge scarcity by assessing the effectiveness of MCS therapy on psychopathology symptoms, psychological distress, and life expectancy among this group. The concentration on self-compassion in the setting of infertility distinguishes it from conventional mindfulness therapies that improve self-awareness and acceptance. MSC becomes especially important in the context of infertility, as people are dealing with significant feelings of shame, guilt, and suffering. MSC is a specialized approach that addresses these emotional difficulties directly. Self-compassion, in addition to being a result of mindfulness, works as an effective mediator for a variety of psychological factors. Through self-compassion, one may cultivate a compassionate and understanding connection with oneself, which aids in reducing the negative impact of fertility-related despair. This program tries to deftly inculcate self-compassion which can function as a mediator, giving participants a comprehensive grasp of its therapeutic function when navigating the complications of infertility. It is hypothesized that the intervention would significantly improve baseline condition and that the effect would endure after treatment (i.e., at the post-treatment and follow-up), and that the intervention group would also benefit psychologically (i.e., reduction of psychological disorder and psychopathological symptoms) more than the group receiving treatment-as-usual (TAU) protocol, resulting in a progressive, significant decrease in psychopathology symptoms and psychological distress levels.

## Methods

### Ethical considerations

This study followed the guidelines outlined in the Helsinki Declaration. All participants were orally informed of the study's anonymity, that their participation was entirely on voluntary grounds, and that they might opt out at any point. Participants were given written informed consent forms. The Ethics Board of Shiraz University of Medical Sciences (registration code: IR.SUMS.REC.1400.311) granted ethical approval. This study was a preregistered trial with the registry code of: IRCT20211118053093N3.

### Study layout

This was a double-blind, two-armed, randomized controlled trial (RCT) with a two-month follow-up that assessed the effectiveness of a program based on compassion-focused therapy, mindful awareness of psychopathology symptoms, and enhancing life expectancy in IW undergoing IVF. Fifty-seven IW were matched for age and then randomly assigned in either an MSC therapy program (*n* = 29) or a TAU protocol (*n* = 28), which offers IW routine psychoeducation and information about infertility and IVF treatment. Power calculations indicated that a sample size of 50 was provided sufficient power (0.80) to detect an effect size of 0.50 (assuming alpha = 0.05, [58]).

### Randomization & blinding

**Fig. 1:**
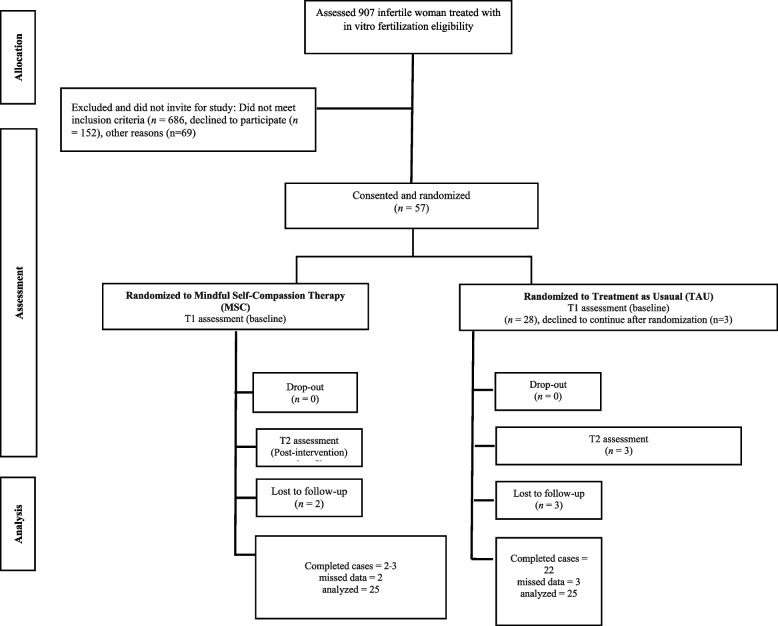
Recruitment and Consort flow of participants through the study

In the randomization process, 57 individuals aged 18 to 45, diagnosed with infertility and undergoing IVF, were evenly distributed into two groups. This allocation was achieved through a 1:1 assignment proportion, utilizing a software-generated random-sampling strategy in Microsoft Excel (2018) to create unique codes for every participant [59]. The first 29 participants were assigned to the MSC therapy arm, while the subsequent 28 were allocated to the TAU group (refer to Fig. 1). Allocation concealment is maintained, with the randomized sequence list and group allocation remaining undisclosed to the research team until the registration phase concludes. Throughout the study, an impartial research assistant, blind to the patients' treatment status, administered all assessment tools. These assessments, covering psychopathology symptoms, were conducted at the beginning of treatment, the conclusion of the treatment, and at the two-month follow-up. Blinding procedures extended to all re-evaluators during follow-ups to maintain the integrity of the study. The individuals responsible for reassessing participants at follow-up stage were also kept blind to the patients' treatment status. This ensures an unbiased evaluation of outcomes throughout the study period.

### Participants & participation processes

Participants were referred to Shiraz Infertility Center for treatment and were receiving IVF treatment. The research was conducted at the Infertility Research Center, in Shiraz, Iran, from August 2021 to June 2022. Participants were not paid any remuneration for being involved in this study. However, their husbands were offered two free sessions of stress management and coping with the IVF situation at the end of the program. The Shiraz University's ethical committee granted permission to conduct human subjects research. The following criteria were used to determine eligibility: having at least a diploma level of education, one year after a definitive diagnosis of infertility, no chronic debilitating physical ailment, and no psychiatric disorder that required prescription of psychiatric medicines before or during the trial (stated by the patient and evidenced by the medical file). Women who missed two sessions, got sickness while participating in the research, attended another training or treatment workshop, or refused to continue cooperating were excluded. We experienced a haigh rate of exclusion due to mentioned reasons and also out of privacy considerations (strong cultural streotypes), some individuals chose not to include their spouses in the sessions. This decision was driven either by a desire to maintain confidentiality regarding their utilization of infertility treatment services or by personal preferences that led them to abstain from participating in the sessions themselves. Figure 1 shows the Consort flow diagram. A qualified MSC trainer (Centre for Mindfulness and Medicine in Shiraz, Iran) led the MSC program with over five years of clinical mindfulness research and practice in the MSC paradigm. The supervising clinical psychologist (MHA) evaluated voice recordings from each session to ensure that the treatment protocol was followed and delivered regular feedback to the presenter. At the time of recruitment, pretreatment baseline/screening examinations were performed. Post-intervention evaluations were carried out immediately following the intervention. Follow-up evaluations were conducted two months following the final MSC session. In relation to the sessions, participants being organized to partake in the MSC sessions on a predetermined date subsequent to the IVF sessions. Following the IVF sessions, the department secretary actively communicated with participants to streamline their involvement in the scheduled MSC session. These sessions unfolded within the conference room of Hazrat Zainab Hospital in Shiraz, ensuring a designated and supportive setting for the intervention.

### The mindful self-compassion program session details

A session-by-session publication version of the MSC protocol [57] was used (see Table 1). Each session concentrates on a particular point. The first session includes a debut, overview, and discussion on self-compassion. Session two covers the fundamentals of mindfulness, including how our mind can seek problems (solving) directed to the future or past. Session three focuses on the use of self-compassion across different parts of life. Week four assists individuals in developing a compassionate inner voice. Session five addresses the importance of living according to core values as well as practicing self-compassion to deal with emotions about life that did not serve values so far. Session six discusses techniques for coping with challenging emotions. Session seven focuses on coping with complicated experiences of interpersonal interactions. During 7th session, participants acquire skills in employing compassionate expressions to manage complexities within challenging relationships, alongside engaging in exercises focusing on mindful breathing. The last (8th) session focuses on the ways of recognizing positive aspects of the self and the individual's life. Between sessions four and five, there is a half-day retreat in which four hours are spent in solitude, performing meditations, restorative yoga, and mindful eating. Each MSC sessions lasts two hours. At this stage of the study, the TAU group received the established procedure. But for ethical considerations, a 2-h session informing about IVF was held at the completion. For both groups, a pre-test was administered before the start of the first session, and a post-test was administered at the completion of the last session. The follow-up was done two months later.

**Table 1:** Mindful Self-Compassion Intervention Protocol

Session 1	Exploration of Mindful Self-Compassion: Participants establish connections and discuss the goals of the therapeutic program during this interactive session. Foundational knowledge is provided by concepts such as self-compassion, mindfulness, self-kindness, and shared human experience. Individuals learn about these ideas' transforming potential via discussion and education, which builds a feeling of belonging and creates the conditions for cooperative personal development
Session 2	Exercise in Mindfulness: Participants delve into the study of mindfulness in this session, uncovering its theoretical foundations as well as its real-world applications. The conversation clarifies the innate propensity of the mind, especially during times of relaxation, to look for problems in the past or project difficulties into the future. In order to achieve this peaceful mental state, participants are led to focus on the here and now, frequently on something as basic as their breathing. By means of this deliberate practice, people are able to access the metamorphic potential of mindfulness, cultivating a keen awareness that transcends the boundaries of past and future worries and eventually leading to a more peaceful and focused life
Session 3	Exercise in Loving-Kindness Meditation: Using expressions like "May I be safe" to develop self-compassion, participants in this workshop engage in the practice of loving-kindness meditation. This method encourages a constant emphasis on compassion throughout the day, even outside of the session. Reciting affirmations repeatedly each day develops into a potent habit that fosters positive self-engagement and emotional resilience. This deliberate practice of self-compassion helps people both personally and in their relationships with other people. Participants set out on a quest to build a solid foundation of compassion in their daily lives via the repetition of simple phrases
Session 4	Finding Your Compassionate Voice: The primary goal of the fourth session is for participants to practice loving-kindness and compassionate words in order to find and develop their compassionate inner voice. The focus is on developing a tone of self-compassion, which stands in sharp contrast to the inner critic, which frequently takes the form of self-judgment
Session 5	Deep Living: The goal of this session is to explore the fundamental principles that give our life purpose. It also seeks to reveal how self-compassion may help us find comfort when we acknowledge that there are times in our lives when our ideals may not be in line with who we are. By means of this investigation, individuals set out on a quest to recognize and harmonize with the principles that are fundamental to their being, enabling self-compassion to function as a compass in managing obstacles and cultivating a feeling of fulfillment and genuineness
Session 6	Regulationg Difficult Emotions: During this session, participants receive guidance on how to softly tag challenging emotions as mild feelings in order to identify and acknowledge them. The main practice is developing labeling and body awareness abilities via gentleness, acceptance, and tranquility. Through the development of a compassionate approach toward difficult emotions and a nuanced awareness of their emotional experiences, this practice is especially helpful in traversing the complicated terrain of shame
Session 7	Relationship Enhancement: In this session, participants learn to use compassionate phrases to navigate the challenges of difficult relationships and practice mindful breathing. In addition to using mindful breathing as a self-soothing method, participants gain the ability to face the challenges that arise in interactions with others by learning how to practice compassionate endurance. The transforming impact of mindful presence and compassionate communication in improving the resilience and quality of interpersonal relationships is highlighted in this session
Session 8	Recognizing Life's Blessings: The last lesson highlights how the human mind is innately biased toward pessimism. Examining threats to our bodily or mental well-being may be helpful for survival, but it may also interfere with our ability to be happy. The session tries to promote perspective shifting by asking participants to recognize and value the good things in life. Individuals develop an attitude that promotes happiness and thankfulness by acknowledging and appreciating their gifts in life. This counteracts the natural tendency toward negativity and improves their general feeling of well-being

### Treatment As Usual (TAU)

The conventional treatment protocol comprised standard medical procedures for IVF, augmented by limited yet crucial psychological support services extended to all inpatients, including those enrolled in the trial. A dedicated team of social workers played a pivotal role in guiding couples through the intricacies of their diagnosis, hospital procedures, personnel interactions, and daily routines. This holistic approach went beyond the immediate medical context, ensuring that participants were well-informed about outpatient services post-IVF, emphasizing a comprehensive and patient-centered approach. Furthermore, the hospital orchestrated a weekly one-hour unstructured group discussion tailored for couples undergoing IVF, under the guidance of hospital personnel (psychologist). This initiative, while voluntary, was actively encouraged, fostering a sense of community and empathy among participants sharing similar challenges. Importantly, the research staff maintained an impartial stance toward these support services, emphasizing the clear demarcation between the research goals of the study and the hospital's psychosocial support initiatives.

### Measures

The revised 90-Symptom Checklist (SCL-90-R [60]) is a self-administered questionnaire with 90 items designed to assess psychological distress/symptoms. The SCL-90-R assesses psychological distress through the use of nine underlying symptomatology and three aggregate values known as global scores. Phobic anxiety (PHOB), somatization (SOM), paranoid ideation (PAR), psychoticism (PSY), anxiety (ANX), hostility (HOS), interpersonal sensitivity (INT), obsessive–compulsive (OBS), and depression (DEP) are the primary symptom aspects. The Positive Symptom Total (PST), Positive Symptom Distress Index (PSDI), and Global Severity Index (GSI) are the global metrics. Referring to the literature on IVF and considering the emphasized psychological problems, we have adopted four subscales of SCL-90-R in this exploration, including ANX, HOS, INT, and DEP. Psychometric properties of the SCL-90-R were found to be good in the Iranian sample, and Cronbach's alpha coefficients for the subscales ranged from 0.75 to 0.92 [61, 62]. The internal consistency of SCL-90-R in the present study ranged from 0.78 to 0.90.

Snyder's Hope Questionnaire [63] is a 12-item self-report questionnaire that assesses the participant's level of hope incorporating two domains: agency (evaluates one's goal-directed energy to pursue one's aims) and pathway (estimates one's amount of constructing means to attain one's goals). Each question is scored using a 4-point Likert scale ranging from 1 (*strongly disagree*) to 4 (*strongly agree*). As a result, the overall hope scale score varies from 12 to 48, with lower scores indicating less agency and hopelessness and higher scores indicating hope. In the Iranian population, the psychometric properties of the hope scale demonstrated acceptable internal consistency (α = 0.70 – 0.89) [64]. The internal consistency of the questionnaire for the present study was good (α = 0.87).

**Satisfaction with MSC Program.** Two Likert scale items were offered to MSC group participants to assess contentment with the MSC Program: "*What has been your experience with the MSC in terms of improving your emotional responses and your capacity to cope with the current situation*?" and " *Is the MSC program something you suggest to others suffering from similar issues?*" Their satisfaction with the MSC intervention was graded on a 5-point scale ranging from 0 (completely dissatisfied) to 10 (completely satisfied).

### Analyses plan

As part of data screening, consistency checks, and descriptive and graphical analyses were conducted. A comparison was made between the mean and the 5% trimmed mean (*p* > 0.05) to determine whether outliers should be retained or removed. There was no significant difference between the results of the main study when outliers were included or removed. Therefore, data were evaluated using the original data without removing outliers [54, 65].

Using intent-to-treat analysis (ITT), as described in Gupta (2011), the effects of missing data on outcome variables were analyzed by randomly assigning 25 participants to each group [66]. The participant is considered to have withdrawn from the study if more than two intervention sessions have not been attended. Multiple imputations were utilized to estimate missing values (*p* > 0.5; for details, see Table 2). Since the presence or absence of missing data had no effect on the substantive main research findings, therefore, final reports are presented with imputed missing data [67]. To handle missing data, we employed the multiple imputation approach using LISREL version 12 [68]. This software was specifically chosen for its effectiveness in dealing with missing data in our study. We generated a total of 15 datasets to adequately account for missing values across three assessment points for each of the five outcome measures, including hopelessness, anger-hostility, anxiety, interpersonal sensitivity difficulties, and depression. This approach allowed us to obtain robust estimates and effectively address the uncertainty arising from missing data. For the imputation models, we employed unrestricted models, enabling us to capture the relationships and dependencies among variables more comprehensively. By utilizing this approach, we aimed to ensure that the imputation process accounted for the complex interplay among the variables of interest. Furthermore, to assess the missingness mechanism, we conducted Little's MCAR analysis on the dataset. The results indicated that the missing data were completely random (χ^2^ = 30.32, df = 32, *p* = 0.55). This analysis provided support for the assumption of missingness at random (MAR) in our study.

**Table 2:** Comparison of demographic characteristics across groups in both MSC and TAU groups at baseline assessment

	MSC (*n* = 25) *M* (*SD*)	TAU(*n* = 25) *M* (*SD*)	Comparison
Gender [female]	(25)	(25)	χ^2^ (1) = 0.66, n.s
Mean age [years (SD)]	33.36(6.30)	34.72(6.84)	*T* (48) = 0.23, n.s
Educational level [up to12 years at school/ undergraduate degree/ post-graduate degree]	(2/20/3)	(4/17/4)	χ^2^ (2) = 1.05, n.s
Hope (T1)	19.52 (2.50)	20.13 (3.65)	*T* (43) = 0.67, n.s
Anger-hostility (T1)	1.15 (0.41)	0.97 (0.53)	*T* (43) = 1.31, n.s
Anxiety (T1)	1.39 (0.52)	1.22 (0.55)	*T* (43) = 1.08, n.s
Interpersonal sensitivity (T1)	1.21 (0.30)	1.21 (0.33)	*T* (43) = 0.01, n.s
Depression (T1)	3.58 (0.34)	3.63 (0.28)	*T* (43) = 0.54, n.s

Note: *NS* Non-significant

There were 57 IW in the study who underwent IVF. The age range of the participants was 18 to 45 years old (M = 34.04, SD = 6.45). To assess the efficacy of randomization and identify any demographic differences between MSC and TAU groups (Table 2), Chi-square tests and independent t-tests were conducted. No significant between-group differences were found (*p* > 0.05) . Forty-five individuals completed the intervention. There were three TAU group members who could not be contacted for post-test and follow-up assessments (Fig. 1). The two data sets were verified to be matched based on demographic features throughout groups depending on age, gender, educational qualification, hope, anger-hostility, anxiety, interpersonal sensitivity, and depression. Repeated measures of MANCOVAs were conducted to test the outcome variables' differences between the MSC and TAU groups. In all assessment points, a paired t-test was used to compare differences between the MSC group and the TAU group (α = 0.05). Statistical analysis was performed using IBM SPSS v28 [69]. Cohen's *d*, a standardized measure of effect size, signifies the magnitude of the difference between two means. Typically, a value around 0.2 suggests a *small* effect, 0.5 indicates a *medium* effect, and 0.8 or above signifies a *large* effect [70].

### Group (MSC vs. TAU) between-subjects and assessment points

There were three within-subjects tests: pre-intervention (T1), post-intervention (T2), and follow-up (T3). In all assessed domains at T1, there were no statistical differences between the two groups (p > 0.5; for details, see Table 2). A significant interaction between time and group (TIME*GROUP) was discovered. MANCOVAs were conducted for the MSC and TAU groups to measure within-subject effects (TIME), with post hoc pairwise comparisons of variability in mean level at T1, T2, and T3. To evaluate whether there are any differences between MSC and TAU groups in terms of assessed domain mean levels before and after MSC intervention, bootstraps were performed for independent t-tests. A t-test was conducted to compare outcomes for MSC and TAU groups at three different assessment intervals. The Reliable Change Index (RCI; [71]) was applied to explore the clinical significance of five outcomes before and after the MSC intervention.

## Results

MSC group, compared to the TAU group, increases hope and diminishes psychopathologic symptoms in IW undergoing IVF, and these benefits last for two months following the intervention. The repeated measures ANCOVAs were conducted following the finding that there was a significant interaction effect between TIME and GROUP. Results indicated that scores consistently improved in the MSC group with respect to hope, anger-hostility, anxiety, interpersonal sensitivity, and depression from T1 to T3 when compared with the TAU group (Table 3).

**Table 3:** Analyses of repeated measures, plus reliable change index (RCI) scores for hope, anger-hostility, anxiety, interpersonal sensibility, and depression

		**Mean (SD)**						**n (%)**^b^					
**Outcome Domains**	**Group**	**T1**	**T2**	**T3**	Time*group	**Within-subjects**	**Post hoc**^**a**^	**T1** **vs. T2**		**T1** **vs. T3**		**T2** **vs. T3**	
								**RI**	**RT**	**RI**	**RT**	**RI**	**RT**
**Hope**^**c**^	**MSC**	19.52 (2.50)	26.96 (1.94)	26.64 (2.03)	F (2–90) = 87.89, *p* < .001, η^2^ = .66	F (2–48) = 153.75, *p* < .001, η^2^ = .88	T1 < T2 T1 < T3 T2 = T3	22 (88%)	0 (0%)	22 (88%)	0 (0%)	1 (4%)	1 (4%)
	**TAU**	20.13(3.65)	19.54(3.24)	19.09 (2.26)		F (2–42) = 2.04, *P* = .14, η^2^ = .09	T1 = T2 T1 = T3 T2 = T3	0 (0%)	0(0%)	0(0%)	1 (4.5%)	0 (0%)	2 (9%)
**Anger-Hostility**^**c**^	**MSC**	1.15 (0.41)	.39 (0.21)	.49 (0.35)	F (2–90) = 43.42, *p* < .001, η^2^ = .49	F (2–48) = 40.24, *p* < .001, η^2^ = .71	T1 > T2 T1 > T3 T2 = T3	16 (64%)	0 (0%)	14 (56%)	0 (0%)	5 (20%)	12 (48%)
	**TAU**	0.97 (0.53)	1.14 (0.56)	1.10 (0.55)		F (2–42) = 7.16, *P* = .01, η^2^ = .43	T1 < T2 T1 < T3 T2 = T3	0 (0%)	0 (0%)	0 (0%)	1 (4.5%)	0 (0%)	1 (4.5%)
**Anxiety**^**c**^	**MSC**	1.39 (0.52)	0.48 (0.25)	0.53(0.31)	F (2–90) = 35.19, *p* < .001, η^2^ = .44	F (2–48) = 47.97, *p* < .001, η^2^ = .67	T1 > T2 T1 > T3 T2 = T3	17 (68%)	0 (0%)	16 (64%)	0 (0%)	3 (12%)	4 (16%)
	**TAU**	1.22 (0.55)	1.22 (0.59)	1.34 (0.53)		F (2–42) = 1.88, *P* = .17, ns, η^2^ = .06	T1 = T2 T1 = T3 T2 = T3	2 (9%)	1 (4.5%)	0 (0%)	2 (9%)	0 (0%)	2 (9%)
**Interpersonal Sensitivity**^**c**^	**MSC**	1.21 (0.30)	0.62 (0.24)	0.52 (0.31)	F (2–86) = 25.63, *p* < .001, η^2^ = .37	F (2–48) = 44.17, *p* = .001, η^2^ = .64	T1 > T2 T1 > T3 T2 = T3	17 (68%)	0 (0%)	21 (84%)	0 (0%)	9 (36%)	7 (28%)
	**TAU**	1.21 0(.33)	0.97 (0.52)	1.28 (0.44)		F (2–38) = 12.10, *p* < .001, η^2^ = .39	T1 > T2 T1 < T3 T2 < T3	6 (27%)	0(0%)	2 (9%)	2 (9%)	2 (9%)	4 (18%)
**Depression**^**c**^	**MSC**	3.58 (0.34)	1.84 (0.71)	1.97 (0.42)	F (2, 90) = 17.25, *p* < .001, η^2^ = .28	F (2–48) = 64.55, *p* < .001, η^2^ = .73	T1 > T2 T1 > T3 T2 = T3	21 (84%)	0(0%)	18 (72%)	0 (0%)	5 (20%)	7 (28%)
	**TAU**	3.63 (0.28)	3.25 (0.55)	3.06 (0.47)		F (2–42) = 11.92, *p* = .001, η^2^ = .36	T1 > T3 T2 = T3	1 (4.5%)	0 (0%)	7 (32%)	0 (0%)	.7 (32%)	0 (0%)

*T1* pre-test, *T2* post-test, *T3* follow-up; MSC (*n* = 23); TAU (*n* = 22), * *p* < .05), *RI* reliable improvement, *RT* reliable deterioration

^a^Games-Howell post-hoc test for significant homogeneity of variance was used if Leven's test for homogeneity of variance was significant; otherwise, Bonferroni was used; significant pairwise differences are displayed as " > " and non-significant pairs as " = "

^b^The clinically significant changes were calculated using RCI calculations (Jacobson and Traux, 1992)

^c^Confounding impact of age and educational level was controlled as covariate variables

**Fig. 2:**
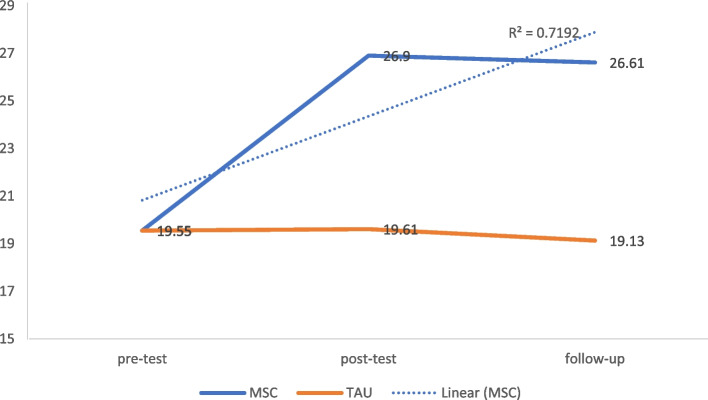
Modified mean score through the assessment time-points for hope after controlling for age and education

**Fig. 3:**
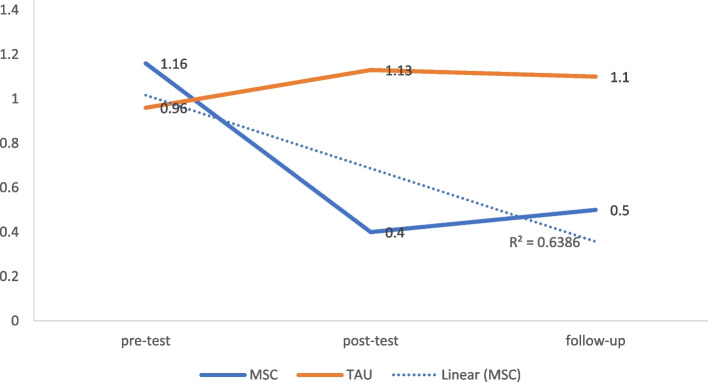
Modified mean score through the assessment time-points for anger-hostility after controlling for age and education

**Fig. 4:**
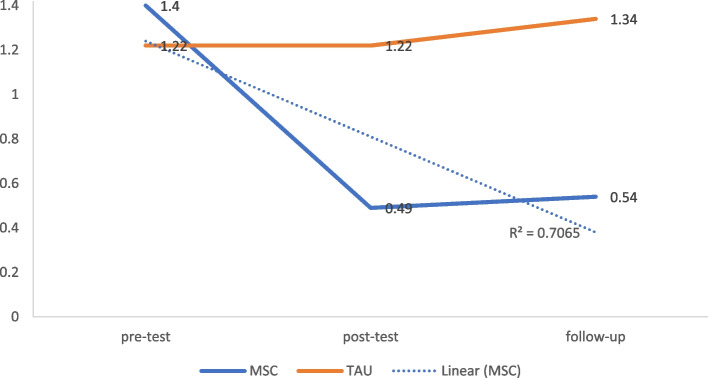
Modified mean score through the assessment time-points for anxiety after controlling for age and education

**Fig. 5:**
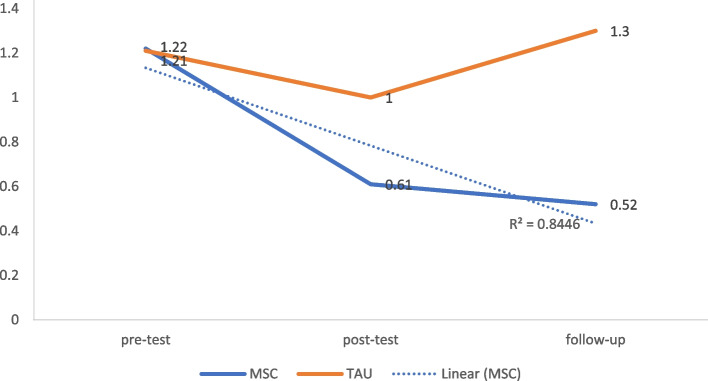
Modified mean score through the assessment time-points for interpersonal sensitivity after controlling for age and education

The mean and standard deviation values presented in the table are prior to controlling for the main effects of gender and educational level. The modified means after controlling are depicted in Figs. 1 to 5

### The hopelessness

Independent t-test bootstraps for MSC and TAU groups at T2 [*t*(45) = 10.06, *p* < 0.001, Cohen’s *d* = 0.85, 95% CI = 0.73 to 0.88], and T3 [*t*(43) = 12.21, *p* < 0.001, Cohen’s *d* = 0.87, 95% CI = 0.80 to 0.91] yielded significant reduction in hopelessness symptoms in the MSC group with a large magnitude of effect (Fig. 2).

### The anger-hostility

A bootstrap analysis of independent t-tests between the MSC and TAU groups at T2 [*t*(43) = 6.34, *p* < 0.001, Cohen’s *d* = 0.68, 95% CI = 0.50 to 0.79], and T3 [*t*(43) = 4.47, *p* < 0.001, Cohen’s *d* = 0.55, 95% CI = 0.32 to .70] showed significant decrease in anger-hostility symptoms in the MSC group with a large effect size (Fig. 3 and Table 3).

### The anxiety

Comparing the MSC and TAU groups at T2 [*t*(43) = 5.48, *p* < 0.001, Cohen’s *d* = 0.63, 95% CI = 0.43 to 0.75], and T3 [*t*(43) = 6.46, *p* < 0.001, Cohen’s *d* = 0.69, 95% CI = 0.52 to 0.79] revealed that a significant reduction in anxiety and phobia symptoms was found in the MSC group with the largest effect size (Fig. 4 and Table 3).

### Interpersonal sensitivity

The comparison of MSC and TAU groups at T2 [*t*(43) = 3.05, *p* < 0.001, Cohen’s *d* = 0.41, 95% CI = 0.14 to 0.60], and T3 [*t*(43) = 36.70, *p* < 0.001, Cohen’s *d* = 0.70, 95% CI = 0.53 to 0.80] revealed a significant decrease in interpersonal sensitivity symptoms in the MSC group with a large effect size (Fig. 5 and Table 3).

### The depression

**Fig. 6:**
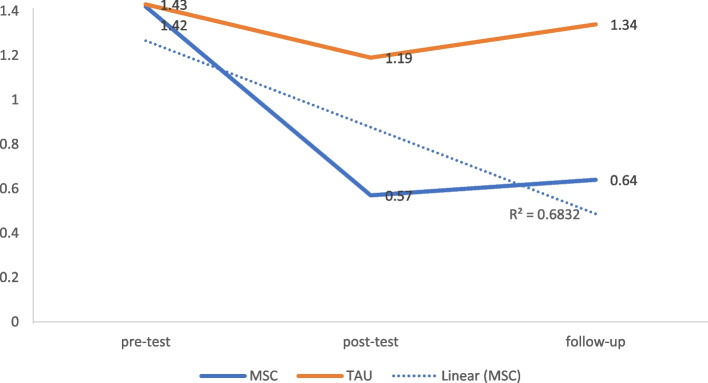
Modified mean score through the assessment time-points for depression after controlling for age and education

An analysis of the MSC and TAU groups at T2 [*t*(43) = 5.21, *p* < 0.001, Cohen’s *d* = 0.61, 95% CI = 0.40 to 0.74], and T3 [*t*(43) = 3.88, *p* < 0.001, Cohen’s *d* = 0.50, 95% CI = 0.25 to 0.66] showed a significant decrease depressive symptoms in the MSC group (Fig. 6 and Table 3).

### A clinically significant change

Results in Table 3 indicate that MSC led to stable changes in the symptoms of hopelessness, anger-hostility, anxiety, depression, and interpersonal sensitivity. At post-treatment (T2), 88%, 64%, 68%, 68%, and 84% of subjects, at follow-up (T3), 88%, 56%, 64%, 84%, and 72% of participants were categorized as reliable improve range for hopelessness, anger-hostility, anxiety, depression, and interpersonal sensitivity, respectively.

### Satisfaction with MSC Program

Participants assessed their satisfaction with the MSC program using a visual analog rating scale ranging from 0 to 10. The results showed that 74.40% of individuals dealing with infertility expressed satisfaction with the program, finding it beneficial for alleviating their negative emotional and psychological symptoms. Additionally, 65.60% of these participants indicated a willingness to recommend the MSC program to others.

## Discussion

The effects of infertility are overarching and may entail negative social effects in addition to individual anguish. Despite obstacles related to medical coverage and expense, advances in assisted reproductive technologies, such as IVF, can bring relief to plenty of couples when treatment is available and viable. Inadvertently, the medical model of infertility has resulted in disdaining from the couples' adverse feelings and emotions, which entails psychological distress, a diminished sense of control, social stigma, and a deviation from the normal course of adulthood growth [12]. Furthermore, self-compassion is a powerful emotion-regulation skill [72]. This points to the necessity for further hands-on research and intervention trials with infertile individuals. According to research, self-compassion can serve as a psychological mechanism that reduces the influence of negative feelings like shame and self-blame on the emotional burden of infertility [53]. We may be able to improve their psychological well-being and potentially even impede the development of mental health difficulties as a result of their infertility experiences if we promote self-compassion in this vulnerable population. Thus, the present study aimed to explore whether MSC therapy might have the potential to reduce psychological distress and symptoms related to mental health in individuals undergoing IVF treatment. We also aimed to understand if it could have a positive influence on life expectancy in this group. Our data showed that women receiving MSC therapy experienced a significant decline in hopelessness. On par with this finding, a large body of research indicates that mindfulness and self-compassion have negative associations with hopelessness in a variety of health (i.e., physical or psychological) conditions, [73–75].

Our findings revealed a significant drop in the anger-hostility index among IVF participants of the MSC program. In accordance with this finding, Morley et al. (2016) have reported that self-compassion plays a buffering role against hostility when individuals are facing adverse life events [76]. One plausible rationale is that self-compassion could potentially discourage individuals from engaging in self-criticism, blame, or censure toward themselves and others when goals remain unmet (for example, the inability to get pregnant) [77]. This can potentially diminish the experience and manifestation of hostile behavior and anger. Consistent with this view, research suggests that self-compassion helps people eschew being judgmental of others and controlling their own and others' emotions, accept their own and others' flaws, and encourage reconciliation in themselves and others [78, 79]. These important qualities of self-compassion may operate as a buffer against the negative demands of the exacting circumstances of the inability to reproduce and also undergo IVF, as well as their consequent feelings of hostility and anger.

The findings regarding anxiety and the MSC program yielded promising results. We observed a significant drop in the anxiety level of patients, which is substantiated by the evidence thus far. A large body of data supports the substantial effect of self-compassion in anxiety spectrum disorders [80–83]. In a relative vein, Makadi and Koszycki (2020) examined the association of mindfulness and self-compassion with social anxiety disorder and other mental health indicators (e.g., depression, life satisfaction, etc.) and noted that individuals with higher levels of mindfulness and self-compassion reported less severe symptoms [84]. They also demonstrated that self-compassion plays a more pivotal role in clinical variables in this context, and the link between self-compassion and social anxiety was mediated by different aspects of mindfulness. According to Gilbert and Miles (2000), self-criticism causes anxiety reactions and might result in blaming if the person feels their critical thoughts are legitimate. Self-compassion is a healthy choice instead of self-defeating behaviors (i.e., self-judgment), and adopting a compassionate and caring attitude toward oneself, particularly when confronted with a social blunder or self-blame, might diminish anxiety and avoidant behavior through its influence on self-criticism and other analogous processes [85].

We found a significant decrease in interpersonal sensitivity among patients in the MSC group. While experiencing social support is a well-established indicator of life quality and mental health, interpersonal sensitivity symptoms may erode this association [86]. Research has found that self-compassion is positively linked with a wide range of advantages in interpersonal relationships (e.g., flexible interpersonal and conjugal functioning, as well as adaptable parenting behaviors, etc.). Accordingly, promoting self-compassion among patients receiving IVF would possibly improve their interpersonal relations (which might be a significant cause of stress and emotional distress due to infertility issues; see [87–89], and in return, it would enhance their interpersonal relationships, which provides a resourceful cycle of positive feedbacks between individual and their social/emotional milieu [90].

Finally, we found that depressive mood and symptomology declined significantly among patients in the MSC program group. The effectiveness of self-compassion-oriented treatment for depression has received a great deal of attention [91], and the findings have been encouraging so far [92]. It has been proposed that self-compassion serves as a repertoire of adaptive emotion-regulation strategies [93] in depressed individuals and is more efficient in regulating emotions at higher depressive states than other strategies, such as reappraisal and acceptance [92, 94, 95]. Adaptive/maladaptive emotion regulation strategies have been found to be critical in depression and other adverse affective states [96–102], and self-compassion as a positive emotion regulation strategy may buffer against other maladaptive emotion regulation strategies such as self-blame or other blame [103], which are highly prevalent among IW undergoing IVF. These findings suggest that MSC therapy reduces psychopathology symptoms and psychological distress and enhances life expectancy in IW undergoing IVF treatment. In the Iranian cultural context, women grappling with infertility often contend with pervasive infertiulity stigma [104], which usualy contributes to feelings of shame and guilt [105]. The MSC program, designed to cultivate compassionate self-behavior, acknowledge shared human experiences, and enhance mindfulness [57], emerges as a potential intervention to alleviate the negative psychological impact of these emotions. MSC has the transformative capacity to reduce self-criticism by providing a perspective that acknowledges the commonality of infertility challenges among many others. It communicates that grappling with profound feelings and emotions is an inherent and shared aspect of the human experience, offering solace to those undergoing such difficulties. This integration may be helpful for women going through IVF since self-compassion can help reduce the feelings of guilt, shame, and distress that are frequently associated with reproductive procedures. The participants' qualitative input revealed enhanced emotional resilience, less self-criticism, and a better capacity to manage the emotional intricacies of in vitro fertilization. In a related RCT, Li et al. (2016) explored the impact of mindfulness-based interventions on quality of life and pregnancy rates in women undergoing IVF for the first time and concluded that awareness of the present moment without judgment aided in better therapeutic interactions, as well as self-compassion and emotion regulation, all of which contributed to higher pregnancy rates and quality of life improvements [106]. The non-pharmacological, short-term (i.e., eight-week) duration of this intervention makes it a safe, viable treatment for IVF recipients. McDowell et al. (2016) conducted a longitudinal research in which 305 IVF women participated in a one-year follow-up survey [107]. They found that several psychological factors, such as secure attachment, social support, mindfulness, and self-compassion, may positively influence the success of IVF treatment.

In terms of participant and therapist compliance, we continually monitored and assessed their engagement throughout the study. Participants in the both therapy group demonstrated impressive compliance, with a high attendance rate at planned treatment sessions and continuous involvement in mindfulness activities between sessions. This level of commitment was notably encouraging, given the mental and physical strains of IVF infertility treatment. In addition, therapists who delivered the intervention adhered strictly to the stated procedure, guaranteeing uniform and standardized administration of mindfulness self-compassion therapy. This dedication was maintained by regular monitoring and fidelity checks, which contributed to the internal validity and reliability of the study. The commitment and cooperation of both participants and therapists were critical to the study's success and the valuable insights gained from our examination.

### Limitations and future research

Despite its positive outcomes, the current study featured a number of limitations. We could only employ a modest sample size and compare the experimental intervention to a static TAU group, potentially limiting the generalizability of the results in comparison to other demographics and other psychological treatments. We were guided by the evidence-based MSC guidelines in clinical settings. However, further study on the MSC protocol in medical contexts is required. Themes of research might include analyzing and adjusting for the possible impacts of different diagnoses or treatment procedures, investigating how these factors impact participants' therapeutic compliance or engagement, and finding ideal ways/populations to deliver MSC. Also, we controlled for age and educational level, recognizing their potential impact on psychological outcomes. Furthermore, to ensure a comprehensive analysis, we suggest for future direction to to encompass various covariates, such as individual differences, contextual variables, and therapeutic elements. By incorporating a range of covariates, we could enhance the robustness of our analysis and facilitate a nuanced interpretation of the observed changes in psychological outcomes—from pre to post-intervention and follow-up.

In this study, we have tried to explore the most accentuated problematic emotions and conditions in IVF literature. Nonetheless, clinging to the literature and extant studies has unintentionally hindered us from having the prerequisites (i.e., all the SCL-90 subscales) to assess the Global Severity Index (GSI) and Positive Symptom Distress Index (PSDI). Future studies can depict a more comprehensive delineation of psychological distress and severity among IVF patients by implementing all the subscales of SCL-90. During our trial, we met some real-world obstacles that highlighted the practical limits of these methods. The significant time commitment necessary for participants to continue a regular mindfulness practice within the framework of self-compassion was one notable barrier. Our experiment showed that, while everyday practice is optimal for the greatest benefits, it proved difficult for many of our paricipants owing to the demands of their daily lives. Mindfulness practices urge individuals to pay attention to their thoughts and emotions (apecifically adverse ones), which might elicit unpleasant or upsetting emotions. The results showed that some participants experienced discomfort when they began initial sessions, with a heightened awareness of uncomfortable thoughts and feelings. This initial uneasiness, if not handled properly, may impede their continuing participation in the therapy. Finally, our outcomes were focused on self-report. Feedback from clinicians who are not informed of the treatment condition may be a more robust indication of improvement than patient reports. Future research may benefit from integrating behavioral, psychophysiological, and neurological examinations [108]. In conclusion, the MSC program's implementation on IW undergoing IVF and its encouraging results demonstrated that it can be a flexible tool in medical settings when medical conditions are accompanied by exacerbating psychological difficulties.

## Data Availability

This study's data can be accessed from the last author upon request. The data is not available publicly due to ethical or privacy concerns.
